# Physiological factors affecting platelet-rich plasma variability in human and veterinary medicine

**DOI:** 10.3389/fvets.2025.1571373

**Published:** 2025-05-21

**Authors:** Rocío Colomer-Selva, Asta Tvarijonavciute, Lorena Franco-Martínez, Ángel María Hernández-Guerra, José María Carrillo, Mónica Rubio, Joaquín Jesús Sopena, Katy Satué

**Affiliations:** ^1^Bioregenerative Medicine and Applied Surgery Research Group, Department of Animal Medicine and Surgery, CEU Cardenal Herrera University, CEU Universities, Valencia, Spain; ^2^Interdisciplinary Laboratory of Clinical Analysis (Interlab-UMU), Regional Campus of International Excellence “Campus MareNostrum”, University of Murcia, Murcia, Spain; ^3^García Cugat Foundation CEU-UCH Chair of Medicine and Regenerative Surgery, CEU Cardenal Herrera University, CEU Universities, Valencia, Spain

**Keywords:** PRP, platelet-rich plasma, growth factors, age, sex, BMI, regenerative medicine

## Abstract

Platelet-rich plasma (PRP) is the fraction of autologous plasma that contains a supraphysiological concentration of platelets, which are an important reservoir of growth factors. PRP is today one of the treatments of choice in regenerative medicine in both humans and animals. The aim of this study was to assess the existing literature on the possible influence of the patient’s physiological factors on different PRP characteristics, like platelet concentration, quantity of obtained plasma, quantity and types of growth factors (GFs) in human and veterinary medicines. In addition, a secondary aim was to compare protocols used to prepare PRP in these studies. In a total of 34 studies evaluated, the use of different protocols for the preparation and characterization of PRP and certain physiological factors such as age, sex and body condition substantially modify the characteristics of PRP in both human and veterinary medicine. Therefore, it is essential to standardize PRP collection protocols and define study groups based on physiological factors like sex, age, body mass index, fertility status and analyzed GFs to ensure reliable data and derive precise, informative conclusions.

## 1 Introduction

Platelet-rich plasma (PRP) is the fraction of autologous plasma obtained by centrifugation from whole blood (WB) that contains a supraphysiological concentration of platelets (PLT) (1). To date, there is no standardized protocol for obtaining PRP, and the composition of different PLT concentrates varies in terms of PLT, red blood cell and white blood cell content as well as growth factors (GFs) and fibrin (2). Nevertheless, based on fibrin and cell composition, PRP products are classified into four groups: Pure Platelet-Rich Plasma (P-PRP), Leukocyte- and Platelet-Rich Plasma (LR-PRP), Pure Platelet-Rich Fibrin (P-PRF), and Platelet-Rich Fibrin (3). PLTs are an important reservoir of GFs, such as platelet-derived growth factor (PDGF), transforming growth factor-β1 (TGF-β1), transforming growth factor-β2 (TGF-β2), vascular endothelial growth factor (VEGF), basic fibroblastic growth factor (bFGF), insulin growth factor (IGF), hepatic growth factor (HGF) and epidermal growth factor (EGF). These GFs are biologically active polypeptides involved in chemotaxis, migration, cell proliferation, differentiation, angiogenesis, and extracellular matrix synthesis (4, 5). As de Marcos et al. (6) affirm due to its easy preparation, good tolerance and minimal complications, PRP is today an attractive treatment in human and veterinary medicine.

PRP applications are highly widespread in diverse regenerative fields, including dentistry, orthopedic, ophthalmology, dermatology and gynecology, but there are still no guidelines on the best protocol for PRP preparation and administration (7, 8). Indeed, there are a variety of factors such as the volume of blood sample drawn, the type of anticoagulant, the gravitational force and duration of centrifugation and the use and type of PLT activator that could lead to variations in results in human (8) and veterinary medicine (6). However, besides of the methodologies-related factors, other factors related to the age and sex of the patient contribute to variations in the PLT count, PLT distribution width (PDW) and the mean platelet volume (MPV) among others (9). These variations could contribute to inconsistencies in the composition of PRP and the concentration of GFs, providing variability in the regenerative and healing effects on the tissue to be treated.

This review aimed to assess the existing literature on the possible influence of the patient’s physiological factors on different PRP characteristics, such as age, sex, body mass index (BMI) or fertility status, among others, may affect the characteristics of the obtained PRP, including PLT concentration, GFs concentrations, or expected therapeutic effect in human and veterinary medicines. In addition, a secondary aim was to compare protocols used to prepare PRP in these studies and to highlight interspecies differences and inform future protocol development across contexts.

## 2 Materials and methods

The inclusion criteria were defined based on population, concept, and context. Regarding population, publications related to either human or veterinary medicine were included, provided they linked platelet concentrates with one or more physiological factors of the donors and described, to some extent, the preparation protocol. Both experimental and clinical studies involving populations with multiple variables were considered. No contact was made with the authors of the studies to obtain additional data.

**Figure 1:**
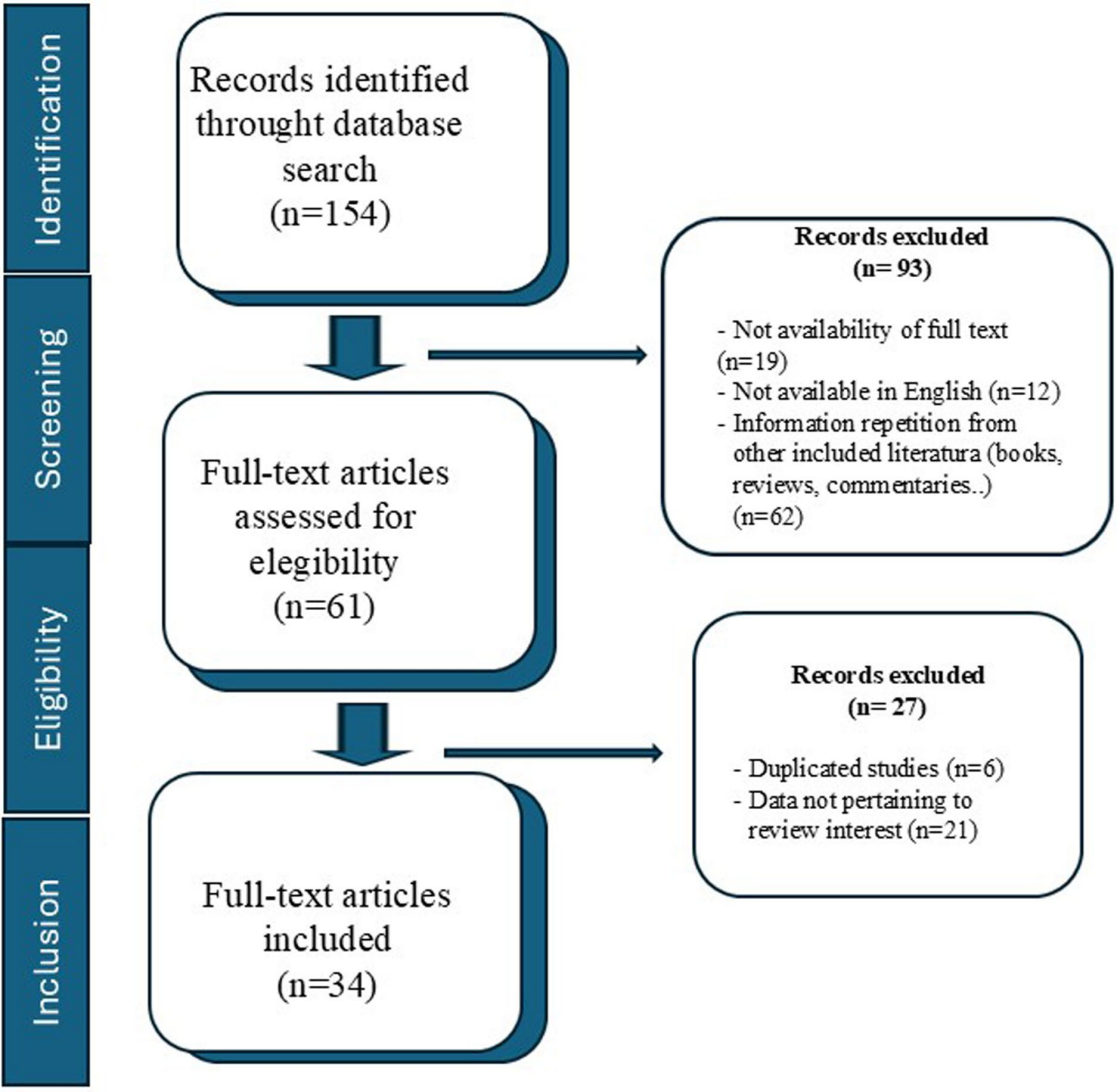
Flow diagram of the search strategy undertaken in the review.

The bibliographic search was carried out in three databases: PubMed, Scopus and Medline databases. Searches were conducted in October 2023, November 2023, and January 2024, with a final search performed in April 2024, limiting the search period between 2013 and April 2024. The search strategy consisted of the use of keywords “platelet-rich plasma” AND (“canine” OR “dogs”) AND (“feline” OR “cats”) AND (“human”) AND “classification” AND “patient age” AND “patient sex” AND “pure-breed” AND “body mass index” AND “height.” Inclusion criteria included all articles containing information on the method of obtaining PRP, centrifugation method (velocity, time, and temperature), type of activator, PLT concentrate or GFs (EGF; bFGF; HGF; PDGF-AA, PDGF-BB, PDGF-AB, TGF-β1, IGF-I or VEGF) were included. Special attention was paid to studies that included physiological characteristics of patients such as breed, age, sex, body condition (BCS), and weight. We also included articles found in article references from the first search. Studies written in different languages from English, not found with full text or had different scopes were excluded, as well as publications which did not correlate with physiological factors or that were review articles. The data obtained from the included articles were recorded in a spreadsheet (Microsoft Excel), where author(s), publication year, study groups, PRP parameters analyzed, species, detailed collection protocol (whole blood drawn, amount of PRP obtained, centrifugation protocol, activator, anticoagulant) and relevant results were documented. Figure 1 shows the tracking of the information search.

## 3 Results

The search strategy yielded a total of 154 articles from the three databases. After the screening, 93 articles were excluded for being books, commentaries or reviews (*n* = 62), written in different languages from English (*n* = 12), not found with full text (*n* = 19). In the second revision, other 27 articles had been withdrawn because did not meet inclusion criteria; 6 were duplicated studies and the remaining 21 provided data unrelated to the review interest. Thus, finally, 34 studies were included for the analysis in this review. Out of these, 15 articles evaluated specific physiological factors with respect to the composition of PRP (9 articles in humans and 6 in animal species).

Studies assessing relation between physiological factors and PRP characteristics in humans are summarized in Table 1. The most studied physiological factors in humans were age (8 articles) and gender (8 articles), and 1 report described the effect of BMI. In relation to PRP characteristics evaluated, most of the studies focussed on PDGF-BB (7 articles), followed by TGF-β1 (6 articles), and IGF-1 and PLT count (5 articles each). Most studies on the human species found a negative correlation between age and the concentration of some of the analyzed growth factors, primarily PDGF-BB and IGF-1 (4, 5, 10–12); while Weibrich et al. (13) did not find any correlation and Rossi et al. (1) only found highest PLT amount in younger patients (<40 years-old). Regarding sex, Evansson et al. (11) reported an increase in certain GFs (EGF, HGF, IGF-1, PDGF-BB) in women, whereas the study by Xiong et al. (12) found opposite results; observing an increase in GFs (PDGF-BB, VEGF, TGF-*β* 1, IGF-1) in men. Meanwhile, Verma et al. (9) reported an increase in platelet distribution width (PDW) in women, while Trevissón et al. (14) argued that age is a more determining factor than gender. Lastly, Rossi and colleagues (1) found no correlation between gender or BMI and platelet concentration in platelet-rich plasma (PRP).

**Table 1:** Studies assessing relationship between physiological factors and PRP characteristics in humans.

Reference	Study groups	Product obtained	PRP parameters analyzed	Main conclusions
Weibrich et al. (13)	Age: 9 age-groups (17–62 years) Sex: W, M	L-PRP	GFs: PDGF-BB, PDGF-AB, TGF-β1, TGF-β2, IGF-1	Age and gender have no influence in PLT count or GFs concentrations
Cho et al. (4)	Age: 24–29, 30–39, 40–49, 50–59, 60–68 years Sex: W, M	L-PRP	PLT count GFs: PDGF-BB, PDGF-AB, PDGF-AA, TGF-β1, IGF-1, FGF-b, VEGF	Intra-individual variations in GFs Positive correlation between TGF-β1, PDGFs and FGF-b and WB and PLT counts; Negative correlation between age and PDGF-BB and IGF-1
Dragoo et al. (10)	Age: 18–30, 30–40, 40–50, 50–60 years Sex: W, M	PRP (NS)	GFs: PDGF-BB, TGF-β1, IGF-1, FGFb, VEGF, IGFBP-2, IGFBP-3	Subjects <30 had higher levels of PDGF-BB, IGF-1 and IGFBP-3 than resting age-groups
Evanson et al. (11)	Age: <25, >25 years Sex: W, M	P-PRP	PLT count GFs: PDGF-BB, PDGF-AB, TGF-β1, IGF-1, EGF, HGF	Age and gender influences PRP’s GFs: Higher EGF, HGF, IGF-1, PDGF-BB in F; Higher EGF, IGF-1, PDGF-AB, PDGF-BB, and TGF-β1 in <25-year-old group
Xiong et al. (12)	Age:18–30, 45–60 years Sex: W, M	P-PRP	Cytokines: IL-1β, IRAP, TNF-β GFs: PDGF-BB, VEGF, TGF-β 1, IGF-1	Age: decreased IGF-1 in older patient’s PRP Gender: increased of cytokines and GFs in males´ PRP
Taniguchi et al. (5)	Age: 20–29, 30–39, 40–49 years Sex: W, M	L-PRP	PLT count GFs: PDGF-BB, TGF-β1, IGF-1, EGF, HGF, FGF, VEGF	GFs: PDGF-BB and IGF-1 decrease with age Positive correlation between four PDGF-BB, TGF-β1, EGF and HGF and platelet counts in PRP
Verma et al. (9)	Sex: W, M	P-PRP	PDW, MPV GF: PDGF-BB	PDW differed significantly with sex, being higher in M
Trevissón et al. (14)	Age: <50, > 50 years Sex: W, M	L-PRP	PLT, WBC, NFS, LYMPH and MON count	Age is a more determining factor than sex in PRP
Rossi et al. (1)	Age: <40, 40–60, >61 years Sex: W, M BMI: <24.9, 25–29.9, >30	P-PRP L-PRP	PLT count	Patients <40 presented highest PLT amount No relationship between gender and BMI, and PLT count in PRP

W, Woman; M, Man; NS, Not specified; BMI, Body Mass Index.

The analysis of physiological factors affecting PRP characteristics in veterinary species (Table 2), shows breed and sex as the most studied physiological factors (both were studied in 4 articles: 3 in equines, 1 in canine) followed by factor of age (studied in 3 articles: 1 in equine, 1 in canine and 1 in bovine), only one article reported the influence of the reproductive status on the bovine PRP. The effect of these factors was correlated with PRP PLT count in 6 studies (4 in equines, 1 in canine, 1 in bovine), with WBC count in 4 articles (2 in equines, 1 in canine, 1 in bovine), and with selected GFs (such as PDGF-BB, TGF-B1 and VEGF) in 2 articles (both in equines). Due to the scarcity of publications in veterinary medicine, the results show little consistency across different studies and species. Regarding the effect of breed, only studies in equines have evaluated this factor, finding differences in PLTs in PRP among different horse breeds (15–17). However, only Giraldo et al. (15) reported differences in PDGF-BB levels among breeds. Regarding sex, only Giraldo et al. (15) found an increase in PRP PLTs and PDGF-BB levels in females; however, this correlation was not found by Miranda et al. (16) in equines or by de Marcos et al. (6) in canines. Regarding the effect of age in veterinary medicine studies, de Marcos et al. (6) found no correlation between age and platelet concentration in PRP from canines. However, Lange-Consiglio et al. (18) reported a negative correlation between age and platelet concentration in PRP from bovids, similar to the findings of Paz et al. (17) and Giraldo et al. (15) in equids. On the other hand, Rinovatti et al. (19) observed an increase in PRP PLTs in horses treated with non-steroidal anti-inflammatory drugs (NSAIDs). Additionally, Lange-Consiglio et al. (18) reported higher PRP PLTS from non-pregnant cows, those with a calving interval of less than 3 months, or when blood was drawn from the mammary vein. Table 3 summarizes the specific correlations reported between the effect of each physiological factor and the composition (number of platelets) of the final PRP.

**Table 2:** Studies assessing the relationship between physiological factors and PRP characteristics in veterinary species.

Species	Study groups	Product obtained	PRP parameters analyzed	Conclusions	Reference
Equine	Time of day; dehydration. overhydration; NSAID therapy; exercise	L-PRP	PLT and WBC counts	Higher PLT count in NSAID treated horses; Increase of WBC count in PRP after exposure to all factors	Rinnovati et al. (19)
Equine	Sex: M, F Breed: ACH, CCH Age: <5, 5–10, >10 years	P-PRP	PLT and WBC counts GFs: PDGF-BB; TGF-β1	Higher PLT and PDGF-BB in CCH compared to ACH; Higher PLT counts in females. P-PRG derived from CCH in females or young horses presented higher PDGF-BB than P-PRG derived from ACH males or younger horses	Giraldo et al. (15)
Equine	Breed: MM, MU, QH, TB	P-PRP	PLT GF: VEGF	Breed significantly influenced PLT count in PRP. Positive (but weak) correlation between PLT count in WB and PRP. VEGF concentration did not vary with breed or gender	Miranda et al. (16)
Equine	Sex: M, F Breed: TB, BCH, BSH, MH, CB	P-PRP	PLT, MPV, RBC and leukocyte counts	Age and PRP PLT count were negatively correlated. PRP PLT higher in MH than in BSH and similar in other equine breeds.	Paz et al. (17)
Canine	Sex: M, F Reproductive status: neutered /entired Weight: median 39.1 kg Breed: 21 different breeds Age: 10–150 months	L-PRP	PLT and WBC counts	Positive correlation between PLTs number and WBC in PRP. Weight is positively correlated with PRP PLT count and negatively correlated with PRP WBC; Age and gender not modified PRP PLTs and PRP WBC counts	De Marcos et al. (6)
Bovine	Sex: F Reproductive status: entired, 75.6% pregnant Calving interval: ≤3, 4–5; ≥6 months Breed: Frisian breed Age:24–48, 49–72, 73–89, 97–100 months Site of blood collection: jugular vein, mammary vein Number of births: 1–2; 3–4; 5–7	L-PRP	PLT and WBC counts	Positive correlation between PLT in WB and PRP. Higher PRP PLT count in non-pregnant compared to pregnant females; Higher PRP PLT in cows with a calving interval of <3 months; Higher PRP PLT count in samples from mammary vein: Higher PRP PLT in 49–72 months old cow and 3–4 births cows Age and PRP PLT was negatively correlated	Lange-Consiglio et al. (18)

M, Male; F, Female; NSAID, Non-steroidal anti-inflammatory; ACH, Argentinean Creole Horses; CH, Colombian Creole Horses; P- PRG, pure-platelet rich gel; MM, Mangalarga Marchador, MU, Mule; QH, Quarter Horses; TB, Thoroughbreds; BCH, Brazilian Criollo Horses; BSH, Brazilian Sport Horses; MH, Miniature Horses; CB, Crossbred Horses.

**Table 3:** Direction of the reported effect of the physiological factors in different species.

Factor	Species			
	Human	Equine	Canine	Bovine
Age	Negative	Negative	NC	NC
Sex	Positive/NC	Positive	NC	NS
Reproductive status	NS	NS	Fertile Positive	Pregnant Negative
BMI	NC	NS	Negative	NS
Breed	NS	Positive / Negative	NS	NS
Intraindividual variability	Positive	NS	NS	NS
[PLT] WB	Positive	Positive	Positive	Positive
Circadian variations	NC	NS	NS	NS

Correlation with PLT concentration and/or GFs in PRP: Positive, Negative, NC (not correlated), NS (not specified); BMI, body mass index; [PLT] WB (PLT concentration in Whole Blood).

**Figure 2:**
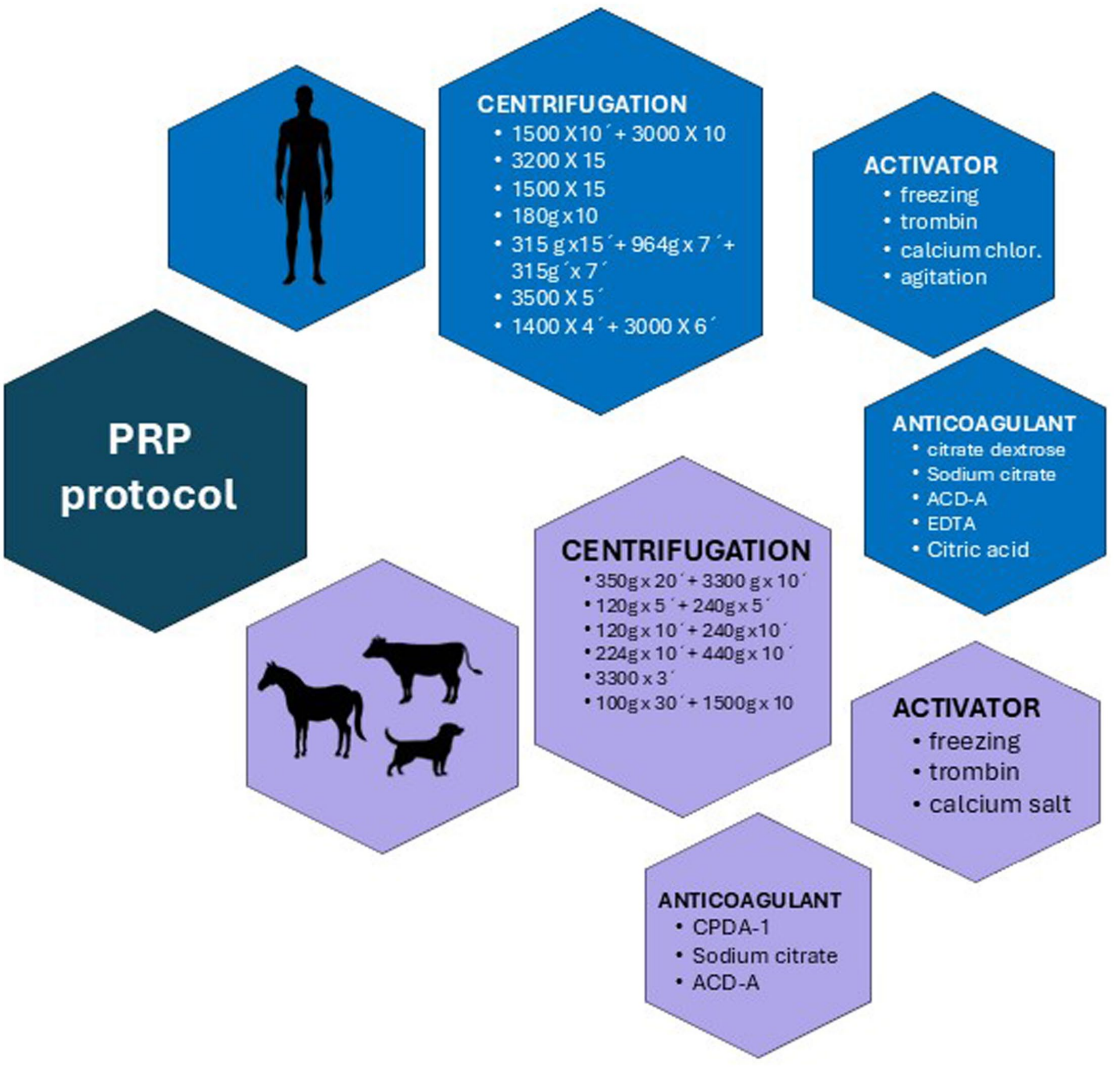
Comparative overview of Platelet-Rich Plasma (PRP) preparation protocols in human and veterinary medicine.

Regarding the protocols used to obtain PRP in the 15 articles in which the possible effects of physiological factors were evaluated, 15 different protocols were used (Table 4). Overall, 10 studies used a double centrifugation protocol and 7 a simple centrifugation protocol: each of them with different velocity and time of centrifugation, different WB volumes and PRP activator; Figure 2 represents differences between protocols. There is a lack of data regarding the PRP collection protocol; founding that 33% of the articles did not specify the platelet activation method, 60% did not clarify the quantity of PRP obtained, and 13% did not mention the anticoagulant used.

**Table 4:** Protocols used for PRP obtaining in human and veterinary medicine to study effects of physiological factors on PRP characteristics.

Species	Centrifugation protocol	WB (mL)	Obtained PRP (mL)	Activator	Antiacoagulant	Reference
Human	NS	400–450	300	Deep freezing	Citrate phosphate dextrose	Weibrich et al. (13)
	Double protocol: 1500 rpm for 10 min and 3,000 rpm for 10 min	9	NS	Bovine thrombin	ACD	Cho et al. (4)
	3,200 rpm for 15 min	55	NS	4 freeze–thaw cycles	Citrate dextrose	Dragoo et al. (10)
	1,500 rpm for 5 min	8,5	NS	Bovine thrombin + 10% calcium chloride	Sodium citrate	Evanson et al. (11)
	180 g for 10 min	25	NS	1 freeze–thaw cycle	Citrate dextrose	Xiong et al. (12)
	Double protocol: 580 g for 8 min and 1,000 g for 20 min	9	2	5% calcium chloride	Citric acid	Taniguchi et al. (5)
	Triple protocol: 315 g for 15 min, 964 g for 7 min and 315 g for 7 min	40	5	Agitation	ACD-A	Verma et al. (9)
	3,500 rpm for 5 min	11	NS	NS	EDTA	Trevissón et al. (14)
	Double protocol: 1400 rpm for 4 min and 3,000 rpm for 6 min	150	NS	NS	NS	Rossi et al. (1)
Equine	Double protocol: 350 g for 20 min and 3,300 g for 10 min	450	NS	NS	CPDA-1	Rinnovati et al. (19)
	120 g for 5 min; 240 g for 5 min	8,5	PRG	Thrombin or calcium salt	NS	Giraldo et al. (15)
	120 g for 10 min; 240 g for 10 min	24	NS	1 freeze–thaw cycle	Sodium citrate	Miranda et al. (16)
	224 g for 10 min; 440 g for 10 min	450	10	NS	CPDA	Paz et al. (17)
Canine	3,300 rpm for 3 min	18 (weight > 10Kg), 9 (weight < 10Kg)	NS	NS	ACD-A	De Marcos et al. (6)
Bovine	100 g for 30 min; 1,500 g for 10 min	450 (aliquots 50 mL)	Platelet pellet	3 freeze–thaw cycle	CPDA-1	Lange-Consiglio et al. (18)

M, Male; F, Female; PRG, pure-platelet rich gel; NS, Non specified; NSAID, Non-steroidal anti-inflammatory; CPDA-1, citrate–phosphate-dextrose solution with adenine; ACH, Argentinean Creole Horses; CCH, Colombian Creole Horses; ACD-A, acid citrate dextrose-acetate; MM, Mangalarga Marchador; MU, Mule; QH, Quarter Horses; TB, Thoroughbreds; BCH, Brazilian Criollo Horses; BSH, Brazilian Sport Horses; MH, Miniature Horses; CB, Crossbred Horses.

## 4 Discussion

Platelet-rich plasma is one of the preferred treatments in regenerative medicine, both in human and veterinary medicines (6, 8). In human medicine, PRP is classified as a substance of human origin (SoHo), which has important implications for its clinical applications and standardization. This classification requires strict regulatory oversight to ensure its safety and efficacy. Indeed, the European Medicines and Medical Devices Agency (EMA) has established specific guidelines and regulations for the production, use, and quality control of PRP in humans (20). However, although regulation is less stringent in veterinary medicine, standards are being implemented to improve the quality and safety of PRP treatments. This includes standardizing platelet concentration and growth factor content, as well as minimizing variability in preparation methods. In fact, the Food and Drug Administration (FDA) has reviewed and made decisions on specific products such as PrecisePRP Canine and PrecisePRP Equine, which are PRP products specific to dogs and horses, respectively (21, 22). Recognition of these regulatory frameworks can guide the development of similar standards in veterinary medicine, potentially bridging the gap between research and clinical practice, improving therapeutic outcomes in animals. In veterinary medicine, interspecific differences in the use of PRP are crucial to understanding its efficacy in different species. These differences can inform the development of species-specific protocols and increase PRP’s therapeutic potential. Research in animal models continues to provide valuable information for adapting and improving PRP protocols for different species, ensuring consistent and effective therapeutic outcomes.

**Figure 3:**
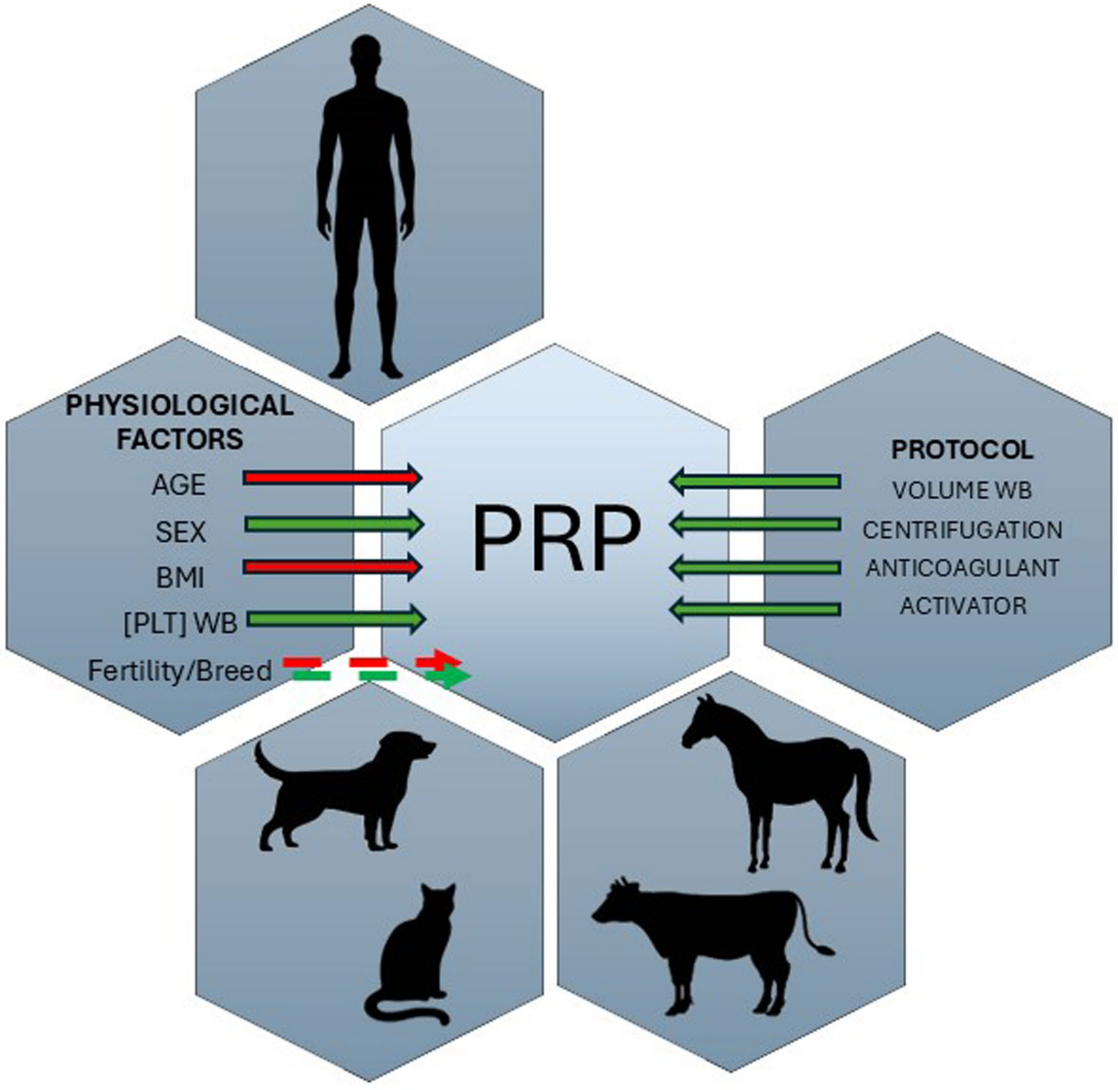
Schematic representation of the physiological and technical factors that influence the final PRP product in both human and veterinary medicine. Physiological factors include age, sex, BMI, fertility status, and breed, while methodological variables encompass whole blood volume, centrifugation settings, anticoagulant choice, and activator use. Together, these parameters significantly affect PRP composition and quality, highlighting the need for species-specific and personalized protocols. The solid arrow indicates direct effects, and the dotted arrow indicates variable effects across studies. The red arrow indicates a negative correlation, and the green arrow indicates a positive correlation.

Besides, numerous factors contribute to variability in the final product obtained, leading to significant unpredictability in the outcomes of its application in clinical practice. This variability arises from both the patient’s intrinsic physiological factors, which influence the quality of the PRP, and the specific protocols used for its preparation (Figure 3). In addition, this variability can also be derived from platelets´ differences in structural and function that have been observed across species, including humans, canines, felines, equines, and bovines. Just for instance, platelets from different species vary in the presence of the surface-connected open canalicular system (OCS), which lacks in some species such cow, camel, and horse (23, 24); also differences in platelet size and morphology have been observed, with variations in granule fusion, cytoskeletal assembly, and membrane spreading among different species (25–27). In relation with functional characteristics, activation and secretion mechanisms differ between species (23), as well as response to thrombin also varies across species (26), at least it has been observed that activity level can be influenced by ecological conditions (28). These differences among platelets call into question the use of the same PRP extraction protocol across different species.

As can be seen throughout the text, the evidence on the possible effect of physiological factors and protocol variations on PRP characteristics is very limited. Furthermore, some of the studies present contradictory results, and most of them do not find a reasonable explanation to discuss their results, which proved to be a constraint for a systematic review and meta-analysis. Nevertheless, this scoping review provides one of the few comprehensive overviews of the literature available in this field and offers some guidelines for future research on the effect that physiological factors (age, sex, BMI, etc.) could have on the PRP obtained.

### 4.1 Age

The influence of age on PLT count on PRP has been evaluated mainly in humans and to a lesser extent in dogs, horses and cows providing contradictory results. In humans, Rossi et al. (1) reported that age (and baseline PLT count) had significant influence in final PRP composition. Furthermore, they detected that age has a significant impact on the PLT count, since for each decade of increase, there was a decrease of approximate 32 k platelets/mm^3^ in the final PRP. In the same line, Hadley et al. (29) reported a negative relationship between age and the number of platelets in WB. However, Weibrich et al. (13) after analyzing 115 samples (stratified by age and sex of the donor) did not observe a significant correlation between age or the previous PLT count in WB and PRP, and the final concentrations of GFs in PRP. On the other hand, Berger et al. (30) observed an age-dependent platelet concentration in older donors (>50 –years-old) but not in young donors (<50-years-old); finding higher PLT concentrations and better dose–response relationship in PRP from older donors.

When a possible relationship between age and GFs in the final PRP was evaluated, Evanson et al. (11) found five GFs, out of seven evaluated, namely EGF, IGF-1, PDGP-AB, PDGF-BB, and TGF-β1, to be significantly higher in people <25-years-old (*n* = 63) than in those >25 –years-old (*n* = 38). Similar results were obtained by Dragoo et al. (10), who, in their study carried out in 40 humans, found higher concentrations of PDGF-BB, IGF-1 and IGFBP-3 in patients below 30 years old. While Xiong et al. (12) detected a decrease in IGF-1 in the group of >50 years-old compared to <50 years-old. In the same way, Cho et al. (4) and Taniguchi et al. (5) reported a decrease in PDGF-BB and IGF-1 with advancing age.

The effect of age on other cellular components of PRP has also been evaluated, reporting a lower monocyte count in women > 50 years and a higher lymphocyte count in men < 50 years (14).

In veterinary medicine, only three articles correlate age with PRP characteristics, one in each species: canines, equines, and bovines. In dogs (*n* = 92), correlation between PLTs, or WBC counts, and age was not detected (6). Similarly, in cows, no correlations were observed between platelet count and age, however, animals between 95 and 120 months-old gave good PRP yields (milliliters of PRP obtained with respect to milliliters of initial blood), and cows aged between 49 and 72 months-old and with 3–4 parities were considered good candidates as PRP donors (18). While Giraldo et al. (15) observed higher PDGF-BB concentrations in pure-platelet rich gel from horses younger than 5-years-old compared to horses older than 5.1-years-old.

Divergent results obtained by different authors could be attributed to the assessment of different populations in terms of age, possible effect of body condition and sex hormones among others together with the use of different protocols and evaluation of different PRP characteristics that together with the relatively low number of articles available, make it impossible to make an accurate conclusion on the age effect on PRP, especially in veterinary species. Despite this, a decrease in the concentration of certain growth factors in PRP, as well as a lower platelet concentration, appears to be observed with increasing donor age in humans. This trend is also seen in equines and bovids but not in canines.

### 4.2 Sex

A contradiction exists on the possible effect of sex in both, human and veterinary medicine. In human medicine, while some authors claim no correlation between PLT count or GFs and sex (1, 5, 10, 13), others report an increase in GFs (PDGF-BB, IGF-1, EGF, HGF), PRP/WBC ratio, PLT, neutrophil and monocyte count in women (11, 14, 29), while another group of authors detected increase in GFs (PDGF-BB, IGF-1, VEGF, TGF-β1) and PDW in men (9, 12). Similarly, in veterinary species, and particularly in horses, PLT and PDGF-BB counts were significantly higher in mares than in stallions (15), while other authors did not find this relationship (16). In dogs, the only existing study assessing the effect of sex on PRP characteristics did not observe possible effect of sex on PLT or WBC counts (6). As a possible explanation for the divergent results among different studies, were indicated hormonal dynamics (15). It is known that sexual steroids, mainly androgens, reduce wound healing in humans and estrogens exert a positive effect on wound healing. This effect could be related to the levels of GFs TGF-b1, IGF-1 and VEGF since estrogens regulate their genetic expression. In fact, in menopausal women, the decrease in estrogens reduces the levels of GFs in the PRP fraction (11).

Therefore, regarding the effect of sex, no clear trend in its influence appears to be observed, with contradictory results found in almost every study included in this review. Three studies found an increase in PLT and GF concentrations in females, while another study reported a decrease in growth factors in females. The remaining studies that analyzed this factor found no correlation.

### 4.3 Reproductive status

To the knowledge of the authors, one study in dogs and one in cows assessed possible relationship between reproductive status and PRP characteristics. In dogs, a significant increase in number of WBC in PRP fractions were reported in non-neutered male population compared to neutered male dogs (6).

In cows, PRP production (milliliters of PRP obtained with respect to milliliters of initial blood) was lower in pregnant than in non-pregnant or postpartum female (18), these differences, about reproductive status have been linked to the higher platelet count in later reproductive states which provides better PRP production (18). Other parameters related to reproductive status such as the reproductive season, the interval between the last birth and blood sampling, the number of births and season of collection have also been evaluated in the bovine species. Indeed, the interval between the last calving (or beginning of lactation) and blood sampling in cows also influences platelet counts. In fact, cows with a calving/cutting interval <3 months have higher platelet yields than those at 4 and 5 months (18). The number of calvings has also been related to the quality of platelet concentrates. In fact, the performance in PRP production in cows with 1–2 and 3–4 calvings was higher than those with 5–7 calvings (18). On the contrary, no differences have been shown in platelet yields in PRP between two consecutive reproductive seasons in this species (18). Finally, the influence of the blood sample collection site on platelet composition has only been evaluated in cows. Blood collection from the mammary vein leads to higher platelet concentrates in PRP than those from the jugular vein (18). Although unknown to these authors, perhaps the lower degree of stress in handling the cows or even the different platelet concentrations in the mammary vein compared to the jugular vein could be related to these differences (18). The only possible influence of the donors’ reproductive status observed in only two studies that analyze this factor is the prevalence of an increase in WBC or PRP platelet concentration in fertile donors.

### 4.4 Body mass index

Regarding the influence of weight in human, Rossi et al. (1) in 357 patients, did not find a significant influence of BMI on final concentration of PLTs in the obtained PRP. However, in veterinary medicine, de Marcos et al. (6) did find a negative correlation between weight and PLT count in dogs, however, this was not specified if weight was dependent on the size of the animal or its body condition.

### 4.5 Veterinary breeds and species

Available results suggest that both breed and species influence platelet count in final PRP. Miranda et al. (16) observed that the platelet content in PRP in Quarter Horses provided higher amounts of platelets, but mules achieved a higher percentage concentration in PRP compared to WB. Likewise, higher platelet concentrations in Miniature Horses compared to Brazilian Sport Horses have been reported by Paz et al. (17). Therefore, species/breed appears to be a factor of variability in the final platelet concentration in PRP, although further studies are needed to confirm these observations in different species/breed of animals.

### 4.6 Other factors

#### 4.6.1 Intra-individual variability

In humans, relatively high intra-individual variability was detected in patients who received two doses of PRP (these patients received 2 doses of PRP separated by 15 days from each other). The comparative study between the first and second dose of PRP among the same patients reported mean counts of 890,018 and 1,244,467/μl, respectively, with a mean difference of 354,448 (1), although in both timepoints same methodologies by the same specialists were used. Overall, these data suggest the need of the in-depth investigation on the repetitiveness of the PRP yield within subject and possible factors that could affect it.

#### 4.6.2 Platelet count in WB

It is a proven fact that platelet concentration in the PRP fraction varies with the platelet count in WB (1, 6, 16, 18). In cows, PRP yield was positively correlated with platelet count but not with WBC in peripheral blood (18).

#### 4.6.3 Circadian variations

In humans, Aoto et al. (31) observed no significant differences in PLT, WBC, RBC or GF counts in PRP samples throughout the day, suggesting that diurnal variation in chronotherapy is not relevant to the use of PRP in clinical practice.

### 4.7 PRP preparation protocol

High variability in the methodologies used to obtain PRP, both in human and veterinary medicine, were detected in the evaluated literature. Main differences were detected in centrifugation protocols and PRP activation methods. These factors significantly affect several quality assessment variables, including preparation method, PRP PLT count and platelet indices such as platelet distribution width (PDW) and mean platelet volume (MPV), contribute to inconsistencies in the composition of PRP, ultimately affecting the GF concentration (32, 33).

Regarding the centrifugation method, some articles express the gravitational force in g and others in rpm, which makes it difficult to compare these different units without knowing the radius length of the centrifugation device. Furthermore, the centrifugation time is variable, ranging between 3 and 20 min.

Likewise, some studies do not include the type of anticoagulant and/or the type of activator. Depending on the collection protocol and factors associated with PLTs such as PDW, MPV and the number of PLTs, they influence the quality of the PLT concentrate and, subsequently, the final concentrations of GFs (7, 9, 34).

Degen et al. (34) compared five different protocols for obtaining PRP in humans, observing that PRP preparations from different separation systems showed similar PLT concentration; however, they demonstrated significant differences in WBC, neutrophil and RBC counts. On the other hand, Mazzocca et al. (35) compared three different protocols in humans, showing differences in PLT and WBC concentrations between the three protocols, they also observed wide variations of intra-individual PLTs and WBC numbers.

Various studies conducted in dogs demonstrate that the PRP preparation protocol affects the characteristics of the final obtained PRP, showing how PLT concentration is not the only important component; neutrophils, mononuclear cells and RBC affects the final product of PRP as well as its clinical efficacy (7, 36, 37).

In relation to PRP preparation protocol in cats, Castilho et al. (38) reported a protocol to produce WBC and PLT-rich fibrin membrane (L-PRF) in cats by centrifuge at 650 g for 12 min. While Miguel-Pastor et al. (2) compared three different centrifugation protocols (255, 260 and 265 g during 10 min) obtaining 1.5 PLT concentration in PRP produced by 10 min centrifugation at 265 g. Alternatively, Chun et al. (39) achieved a significant increase of mean PLT concentration with a double spin system with 3,600 and 3,800 rpm centrifugation. Finally, Ferrari and Schwartz (40) compared two other systems (1,300 rpm and 3,600 + 3,800 rpm) but neither system tested achieved 2–5 times PLT concentration from baseline; they also observed that PLT aggregation presented a significant obstacle to reliable generation of PRP products using feline blood. Most of the articles included in this review agree on the need to standardize a protocol, since this influences the final PRP product obtained.

The study by Franklin and Birdwhistell (41), concluded that the intentional activation of PRP with calcium chloride (CaCl_2_) and thrombin exerts a significant effect on the GF TGF-β1, resulting in higher TGF-β1 concentrations than those obtained in non-activated samples. Furthermore, another study highlighted the importance of the temperature for sample storage as it can affect the final PRP product, since GFs are released at 37°C while they are inhibited at −20°C (42).

The variability found in the PRP extraction protocols is very high, making it difficult to draw conclusions for future studies, beyond the recommendation to record all the details of the extraction protocol, the need for PRP activation prior to its therapeutic application and perhaps an hematological analysis of WB and plasma obtained to assess PLT concentration, as well as the presence of RBC and WBC, would better guide researchers regarding the results obtained in future studies.

## 5 Conclusion

The findings of this review have implications for future studies as understanding the physiological factors that affect PRP and the influence of the selected extraction protocol can improve the design of future research and the interpretation of results. Enhancing the selection of study populations based on these factors, as well as providing detailed descriptions of the selected protocols, is a necessary recommendation for future research. From a clinical perspective, the analysis of donor’s physiological factors like age, sex, BMI, reproductive status, veterinary breed and species, and circadian variations is relevant. Since the PRP effect is based on the concentration of GFs and cytokines contained in the *α* granules of platelets, if the final platelet concentration is altered by these factors, the biological effect on the target tissue will also be modified (1).

Furthermore, it is necessary to standardize PRP collection protocols, define study groups based on physiological factors and the GF analyzed, in order to obtain reliable data and, consequently, draw accurate and informative conclusions. Therefore, future studies with larger and more homogeneous samples are required in relation to sample sizes and distribution by age, gender, BMI, physiological state, etc.; being one of the most important challenges the evaluation of the factors that induce variability based on the clinical characteristics of the patients (13, 43). Homogeneity between results by factor will ensure repeatability and better comparison between studies (44). Therefore, refining research based on these factors is essential to provide consistency and effectiveness in determining which products would be the most indicated and strategic to guarantee their effects in future therapies (45).

The limited scientific evidence on the patient’s physiological factors that may affect the obtained PRP makes it difficult to draw solid conclusions. However, the reviewed studies report variations in PRP characteristics based on specific factors, mainly age and sex, although these findings have not always been consistent across studies. It would also be highly advisable for future research to focus on standardizing an extraction protocol to facilitate the application of scientific evidence in clinical practice. This review provides important insights into the factors that should be considered; enhancing the selection of study populations based on these factors, as well as providing detailed descriptions of the selected protocols, is a necessary recommendation for future research.
